# A pilot study on the effect of feed supplementation with magnesium oxide and magnesium aspartate on blood magnesium content and tail lesions in pigs under practical on-farm conditions

**DOI:** 10.1186/s40813-026-00526-0

**Published:** 2026-06-11

**Authors:** Caroline Jensen, Irena Czycholl, Pauline Nison, Therese Jacquerot, Peter Sandøe, Kathrin Büttner

**Affiliations:** 1 Department of Veterinary and Animal Sciences, University of Copenhagen, Frederiksberg C, 1870 Denmark; 2 VetoAgro Sup, University of Clermont-Ferrand, Clermont-Ferrand, France; 3 Department of Food and Resource Economics, University of Copenhagen, Frederiksberg C, Denmark; 4 Unit for Biomathematics and Data Processing, Faculty 10 - Veterinary Medicine, Justus-Liebig-University Giessen, Giessen, Germany

**Keywords:** Magnesium supplementation, Rearing pigs, Tail biting, Stress management

## Abstract

Tail biting in production pigs is a complex behaviour often triggered by stress and nutritional deficiencies, posing significant welfare and economic challenges. Despite the EU ban on routine tail docking for welfare reasons, docking remains effective in preventing tail biting, which highlights the need for alternative strategies and yet better understanding of the complex mechanisms behind it. This study investigates the efficacy of magnesium supplementation, specifically magnesium oxide (MgO) and magnesium aspartate (MgAsp), in reducing tail biting incidents in pigs postweaning until life week 9 (rearing pigs) with intact tails. Conducted on a Danish farm raising pigs with undocked tails, the trial involved 381 pigs across two batches. Blood magnesium and cortisol concentrations, along with tail and ear lesions, were systematically monitored. Results indicate a decline in blood magnesium levels across all groups after three weeks (i.e. life week 7 of the pigs), coinciding with increased incidences of tail lesions, particularly significant in the group with MgO supplementation. However, discrepancies in magnesium concentrations at the initial time point in the group with MgAsp supplementation suggest potential variations in magnesium absorption or baseline levels unknown prior to supplementation. While MgAsp showed some efficacy in maintaining higher magnesium levels, neither form of magnesium consistently reduced lesion prevalence or stress markers, as indicated by cortisol levels. These findings suggest that while magnesium supplementation can influence magnesium status, its role in mitigating tail biting and associated stress may be limited in the age class of rearing pigs. Further research is necessary to explore magnesium metabolism in young pigs and its intricate relationship with stress, nutrition, and behaviour.

**Supplementary Information:**

The online version contains supplementary material available at 10.1186/s40813-026-00526-0.

## Introduction

Tail biting is a multifactorial behavioural disorder in domestic pigs [40, 52]. Risk factors vary from farm to farm and include genetics, sex, health, neurotransmission, size, growth, age, behavioural phenotype, feeding, space allowance and group size, stockpersons and farm conditions as well as housing conditions and environment (see for an overview on risk factors for example [53]. Tail biting as well as the resulting tail lesions lead to reduced welfare and increased health issues for both biting and bitten pigs [18, 44, 51]. Tail biting also presents economic challenges, including reduced growth, increased labour, veterinary costs, and carcass condemnations [18]. Despite routine tail docking being banned in the EU for welfare reasons, tail docking remains widely practiced as a tail biting prevention method, though it is recognized as a painful procedure [4, 45, 53].

Although risk factors are various [53] as described above and different types of tail biting (as described, for example, by [50] and [53] may require different management intervention strategies, managing stress is crucial for reducing tail biting. Common stressors include regrouping [31], overcrowding [43], and dietary deficiencies [44]. Magnesium supplementation has been proposed as a strategy to reduce stress-induced behaviours in pigs [5]. Magnesium plays a vital role in enzyme function, ATP production, immune regulation, and neurotransmitter activity, including the serotoninergic and adrenergic systems, which may help reduce stress and anxiety [3, 46, 47, 13, 10, 42].

Although several studies report significant improvements in stress markers with magnesium supplementation [2, 31, 35, 36], findings are inconsistent. Some studies suggest improper magnesium dosing may even increase stress [6, 33]. Bioavailable forms such as magnesium aspartate appear more effective, but direct comparisons between magnesium compounds remain limited [5].

In this study, we evaluated various dosages of magnesium oxide (MgO) and magnesium aspartate (MgAsp) with regard to the effectiveness in reducing tail biting in rearing pigs (supplementation started post weaning at 28 days and lasted until life week 9). We monitored blood magnesium and cortisol levels and assessed tail and ear lesions (as outcome-based indicators of tail and ear biting) to determine the efficacy of these supplements. Measuring blood magnesium concentration is a practical approach, as it reflects the animal’s magnesium status and is less invasive compared to tissue sampling. Previous studies have utilized blood magnesium measurements to assess the impact of dietary supplementation on pigs’ stress responses and meat quality [12]. Moreover, [9] found a hypomagnesemia in blood samples of pigs that were analysed with regard to tail biting.

## Materials and methods

### Animals and housing

Data collection was carried out from May to August 2024 in a conventional farrow to finisher pig farm in Denmark raising pigs with undocked tails. The farm kept commercial crossbreeds Duroc x (Landrace x Yorkshire). Male piglets were surgically castrated (with use of analgesic medication and local anaesthesia) during the first three days of life. After a suckling period of 28 days, all weaners were sorted by weight due to the farmer’s management routine. Each group was housed in a unit in the rearing section of the farm, in which typically the pigs are kept postweaning until reaching a weight of ~ 30 kg (in life week 10–11). In total we used five pens for each trial with a maximum number of 50 animals per pen. The pens had part-slatted floors (50%), consisting of concrete with straw bedding. Additionally, the pigs were provided two hands full of fresh straw per day and pen. The space allowance was 0.26 m^2^/pig until time point 2 in our trial (i.e. life week 7) and 0.52 m^2^/pig thereafter. The pigs had multiple enrichment objects in their pen: one lick stone, one wood beam and a plastic toy hanging from the ceiling. These extra provisions enabled the producers to be part of a scheme where the pork products are sold at a premium prize with a national animal welfare label. The pigs were fed ad libitum with commercial dry feed in meal form via an automatic feeder with an animal to feeding place ration of 4:1. This was supplemented with a chicken feeder in the first days to provide more feeding spaces. During the experimental phase, the automatic feeding troughs were filled manually to provide the different added dosages of magnesium supplementation. It was, however, refilled in such a frequency that troughs were never empty, i.e. ad libitum feeding was maintained as usual. Water was available ad libitum via eight nipple drinkers per pen.

### Experimental setup and feed composition

In this study, two different supplementations of magnesium were analysed: supplementation with magnesium oxide (MgO, batch 1) and with magnesium aspartate (MgAsp, batch 2). Within each batch the basic feed was supplemented in different levels with MgO or MgAsp, respectively.

During the rearing phase (postweaning until ~ 30 kg, i.e. life week 10–11), a three-phase feeding system consisting of basic feed and vitamin/mineral feed (Vilomix) was implemented. The composition of the basic feed is shown in the supplementary material (Tables S1 and S2). Table Header from Supplementray Material S2 should include (amino acids) after part 2.

This basic feed was then supplemented with different levels of MgO (batch 1) or MgAsp (batch 2), respectively: within each batch, five different groups with different levels of magnesium supplementation were established. In batch 1 with supplementation of MgO the five groups were as follows: one control group without supplementation and four groups with supplementation of 2, 4, 6 and 8 mg/kg feed of MgO (CALMAGS GmbH, Nutrimag MO 600).

In batch 2 with supplementation of MgAsp the five groups were as follows: one control group without supplementation and four groups with supplementation of 5, 40, 1000 and 2000 mg/kg feed of magnesium aspartate (CALMAGS GmbH, NutriMag AS). In the supplementary material, detailed information of the two supplementations is included in Table S2 and S3.

The above study design could not be exactly fulfilled due to practical reasons. In batch 1, the treatment group supplemented with 8 mg/kg of MgO was removed from the study after the second blood sampling due to farmer’s wishes, resulting in no subsequent lesion assessments or further magnesium and cortisol measurements for this group. Similarly, in batch 2, the treatment group receiving 40 mg/kg of MgAsp was discontinued following the second blood sampling because of farm communication issues. Consequently, data beyond this point are unavailable for these groups, and both groups were excluded from the study.

### Blood samples

As the pigs were newly grouped on day 1 of the rearing phase, to minimise stress on day 1, blood samples for the basal measurement in all three groups were only taken on day 2 of the rearing phase (time point 1, age of the piglets ~ 29 days). Thereafter, blood samples were taken after three (time point 2, age of the pigs ~ 7 weeks) and five (time point 3, age of the pigs ~ 9 weeks) weeks move bracket before weeks to end of sentence. Blood samples were collected following standard veterinary practice. That means, at time point 1 (piglets aged ~ 29 days) and 2 (piglets aged ~ 7 weeks), piglets were turned on their back and blood was extracted from the V. cava cranialis, while at time point 3 (piglets aged ~ 9 weeks), piglets were fixated via sling and blood was extracted from the jugular vein. The whole procedure was carried out minimising stress as far as possible – in particular, in case of high agitation/stress or when blood could not be drawn after a maximum of three attempts, the piglets were released, as the welfare of the pigs was prioritized over the scientific interests in this pilot study. The blood samples were initially cooled, then centrifuged and serum was collected and frozen at -21 °C. After the trial on the farm was concluded, the blood samples were analysed for cortisol and magnesium content at the clinical veterinary laboratory of the University of Copenhagen according to the laboratory’s standard procedures.

### Scoring of tail and ear lesions

Throughout the entire rearing phase, pigs were closely monitored for tail, ear and skin lesions as well as for signs of diseases (such as lameness, coughing and diarrhea) two times a week by an educated observer who systematically recorded all occurrences. In the same recording scheme, the tail posture was recorded. The scoring for tail and ear lesions took place on pen level and the results per pen were recorded as number of animals per pen with as specific scoring. No individual animal tracking was carried out. Table 1 displays the exact sorting criteria for tail and ear lesions. In addition, farm staff monitored the animals with regard to health status and performance in accordance with normal farm routines.

**Table 1:** Scoring system for tail and ear lesions. Adaption from the German scoring key [24, 37]

Score		Description	Summary
Tail lesion	0	No lesion	Skin is intact
	1	Superficial	Points or lines, signs of skin breakage
	2	Small	Deeper skin lesion of small size (max, size = tail diameter at respective location)
	3	Large	Deeper skin lesion of large size, (larger than tail diameter at respective location)
Ear lesion	0	None	Skin is intact
	1	Base	Any lesion (signs of skin breakage, crust or missing part) located at the ear base
	2	Tip	Any lesion (signs of skin breakage, crust or missing part) located at the ear tip

### Final dataset and statistical analysis

After exclusion of one of the feeding groups in each batch (please see “Experimental setup and feeding composition”), in total, 381 animals were monitored throughout the rearing phase (i.e. postweaning until time point 3 (~ life week 9)), divided into two consecutive batches of 191 animals in batch 1 with MgO supplementation and 190 animals in batch 2 with MgAsp supplementation.

Due to the fact that no tracking of the individual animals was carried out each time point was analysed separately. Furthermore, all animals in the pen were scored regarding tail and ear lesions, however, due to practical reasons described above, blood samples were not taken from all animals in the pen. The exact numbers of animals and blood samples taken are illustrated in Table 2 for batch 1 with MgO supplementation and Table 3 for batch 2 with MgAsp supplementation.

**Table 2:** Number of animals per pen and time point as well as the number of blood and cortisol samples taken from each pen and time point for batch 1, the supplementation with magnesium oxide (MgO)

	Time point	Age of the animals	Number of animals	Number of blood samples
Control				
	1	~ 29 days	47	47
	2	~ 7 weeks	46	30
	3	~ 9 weeks	46	42
2 mg/kg				
	1	~ 29 days	45	45
	2	~ 7 weeks	44	31
	3	~ 9 weeks	44	44
4 mg/kg				
	1	~ 29 days	50	50
	2	~ 7 weeks	50	30
	3	~ 9 weeks	50	40
6 mg/kg				
	1	~ 29 days	48	47
	2	~ 7 weeks	48	23
	3	~ 9 weeks	48	36

**Table 3:** Number of animals per pen and time point as well as the number of blood and cortisol samples taken from each pen and time point for batch 2, the supplementation with magnesium aspartate (MgAsp)

	Time point	Average age of the animals	Number of animals	Number of blood samples
Control				
	1	~ 29 days	48	27
	2	~ 7 weeks	48	29
	3	~ 9 weeks	48	23
10 mg/kg				
	1	~ 29 days	50	35
	2	~ 7 weeks	45	18
	3	~ 9 weeks	45	19
1000 mg/kg				
	1	~ 29 days	48	31
	2	~ 7 weeks	47	24
	3	~ 9 weeks	47	24
2000 mg/kg				
	1	~ 29 days	45	29
	2	~ 7 weeks	45	28
	3	~ 9 weeks	45	20

Blood cortisol concentration and magnesium levels were analysed using a one-way analysis of variance, considering the fixed effect of the treatment group. The analyses were carried out separately for each time point. The residuals from this analysis exhibited an approximately normal distribution. P-values for pairwise comparisons were adjusted using the Bonferroni method.

Tail and ear scoring data were evaluated with an exact Pearson Chi-Square Test. Given the limited number of animals exhibiting tail and ear lesions, results were categorized into two groups: tail = 0 (no tail lesion) and tail = 1 (tail lesion, irrespective of severity). Similarly, earlesion data were categorized as ear = 0 (no ear lesion) and ear = 1 (any kind of ear lesion). However, for the figures of the tail lesions the original categorisation of the scoring was maintained.

All statistical analyses were performed using SAS statistical software[39].

## Results

### Blood magnesium concentration

**Fig. 1:**
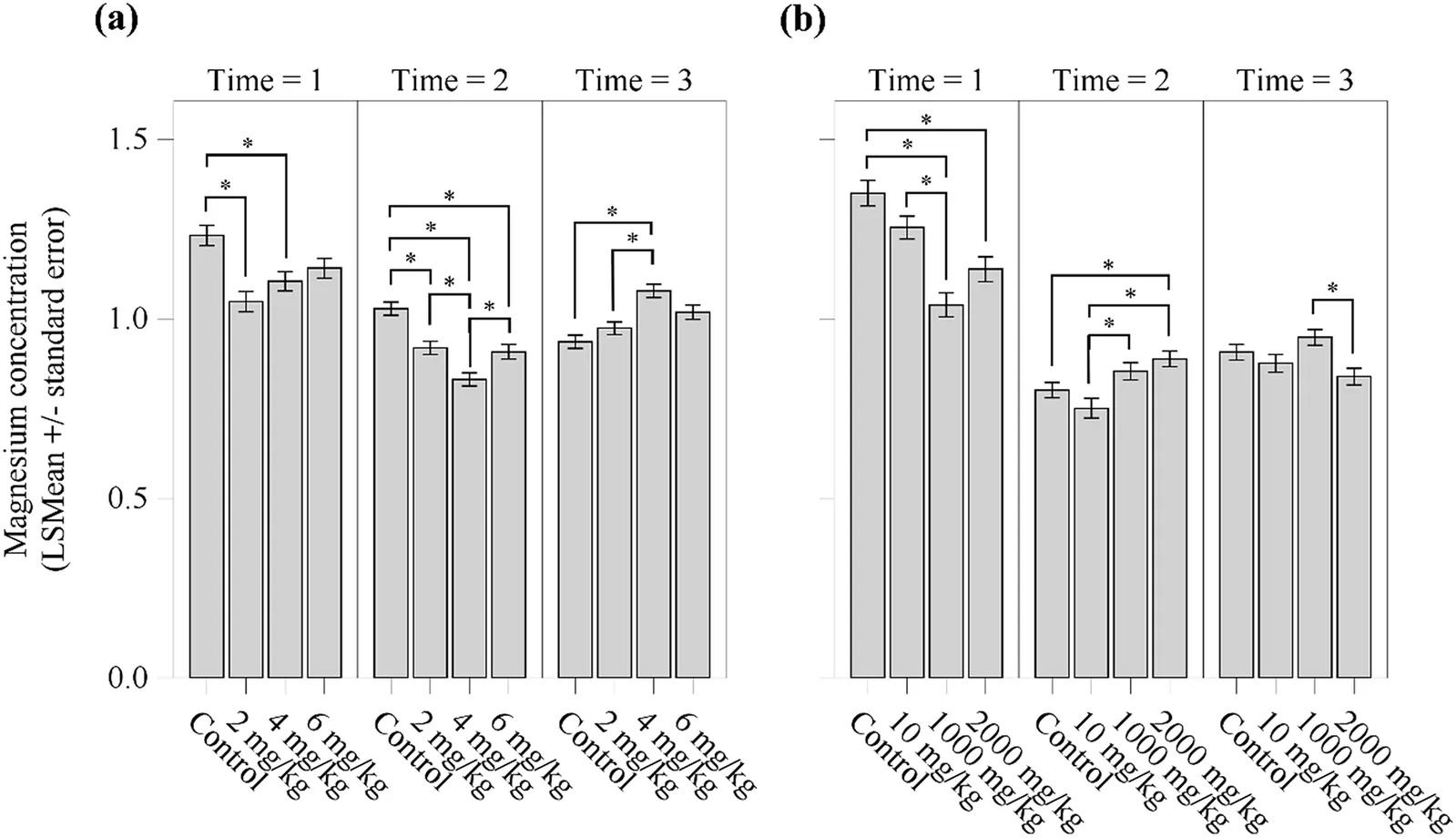
Blood magnesium concentration (LSMeans +/- Standard error) for the two different supplementations, magnesium oxide (MgO, **a**) and magnesium aspartate (MgAsp, **b**) for the different treatment groups and time points (time point 1: age of the pigs ~ 29 days, time point 2: age of the pigs ~ 7 weeks, time point 3: age of the pigs ~ 9 weeks). Significant differences (*p* < 0.05) within each time point between the different treatment groups after Bonferroni adjustment are illustrated by an asterisk

Figure 1 illustrates the blood magnesium concentrations across various supplementation levels, treatment groups, and time points. In both batches, time point 1 displayed the highest blood magnesium concentrations, followed by a decline at time point 2 and a slight increase at time point 3.

In batch 1, with MgO supplementation, significant differences among the treatment groups were observed at all time points (time point 1: *p* < 0.0001, time point 2: *p* < 0.0001, time point 3: *p* < 0.0001). The significances of these pairwise comparisons are detailed in Fig. 1a. No systematic pattern was evident when comparing the different treatment groups.

Also, in batch 2, where MgAsp was supplemented, significant differences between the treatment groups were detected at all time points (time point 1: *p* < 0.0001, time point 2: *p* = 0.0010, time point 3: *p* = 0.0082). Again, the significances for these pairwise comparisons are provided in Fig. 1b.

### Blood cortisol concentration

**Fig. 2:**
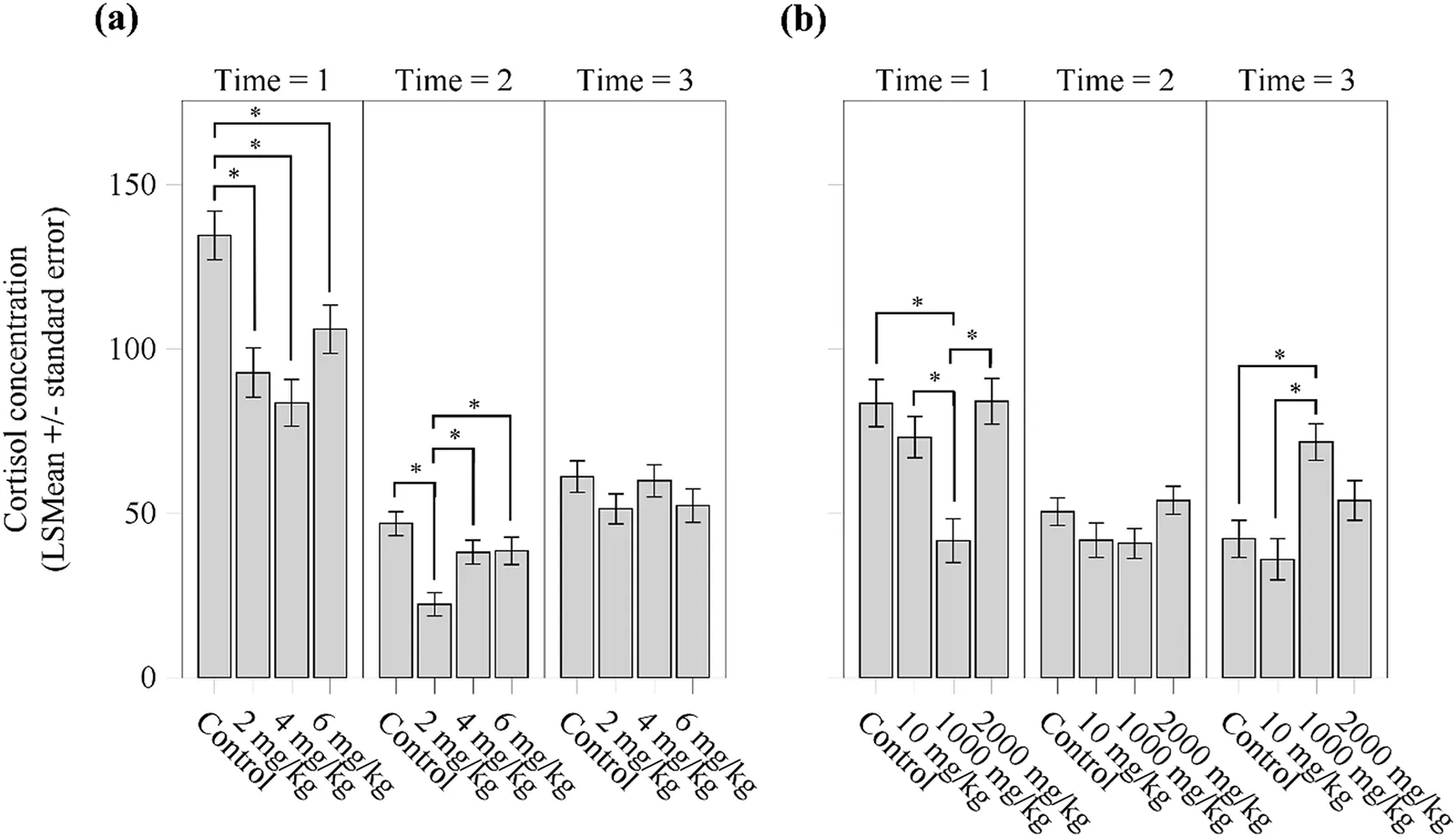
Blood cortisol concentration (LSMeans +/- standard error) for the two different supplementations, magnesium oxide (MgO, **a**) and magnesium aspartate (MgAsp, **b**) for the different treatment groups and time points (time point 1: age of the pigs ~ 29 days, time point 2: age of the pigs ~ 7 weeks, time point 3: age of the pigs ~ 9 weeks). Significant differences (*p* < 0.05) within each time point between the different treatment groups after Bonferroni adjustment are illustrated by an asterisk

Figure 2 illustrates the blood cortisol concentrations at various supplementation levels, treatment groups, and time points. In batch 1, the supplementation with MgO, blood cortisol concentrations peaked at time point 1, decreased at time point 2, and then increased again at time point 3. For batch 2, i.e. the supplementation with MgAsp, no such clear pattern could be detected.

In batch 1, which involved MgO supplementation, significant differences between the treatment groups were observed at time point 1 (*p* < 0.0001) and time point 2 (*p* < 0.0001), but not at time point 3 (*p* = 0.3955). The significances for these pairwise comparisons are detailed in Fig. 2a. No systematic pattern emerged when comparing the different treatment groups.

In batch 2, with MgAsp supplementation, significant differences between the treatment groups were noted at time point 1 (*p* < 0.0001) and time point 3 (*p* = 0.0002), but not at time point 2 (*p* = 0.1171). The significance of these pairwise comparisons is presented in Fig. 2b.

### Tail and ear lesions for the supplementation with MgO

**Fig. 3:**
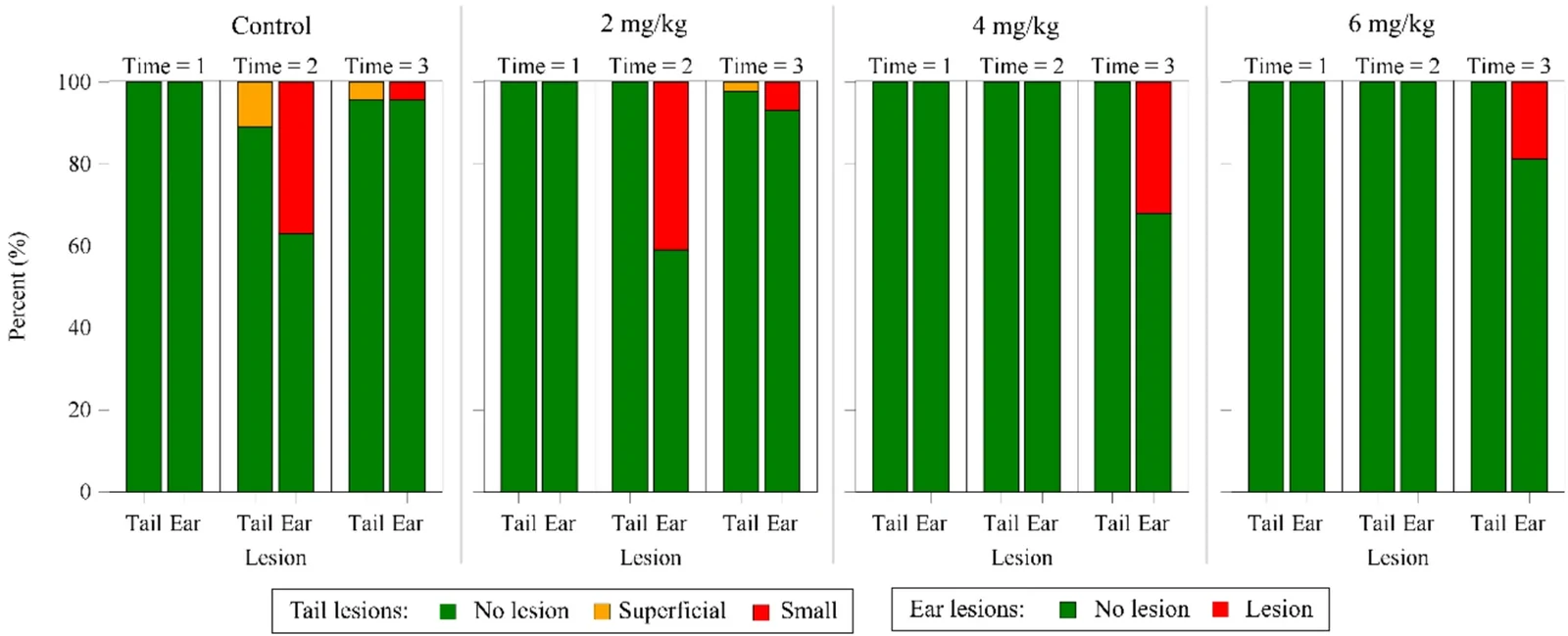
Tail and ear lesions (%) for the supplementation with magnesium oxide (MgO) for the different treatment groups and time points (time point 1: age of the pigs ~ 29 days, time point 2: age of the pigs ~ 7 weeks, time point 3: age of the pigs ~ 9 weeks). Although for statistical analyses tail lesions were binary categorised in no tail lesions and tail lesions, for the descriptive statistic the categorisation no tail lesions, superficial lesions and small lesions was chosen

Figure 3 depicts the percentage of pigs with or without tail or ear lesions across three time points and the different treatment groups for the supplementation with MgO. Over all treatment groups the amount of tail biting was very low, with a maximum of 11% of superficial tail lesions in the control group at time point 2. Furthermore, all tail lesions which were recorded for batch 1 fell in the category superficial lesion. At time point 1, tail or ear lesions could be detected for all treatment groups. Furthermore, treatment groups 4 mg/kg and 6 mg/kg showed also no tail lesions in time points 2 and 3. Within each time point, no significant differences between the treatment groups could be detected.

Similar to the tail lesions, at time point 1 no ear lesions could be detected for all treatment groups. However, contrarily to the tail lesions, more animals were affected by ear lesions at time point 2 and 3. At time point 2, the largest amounts of ear lesions could be detected for the control group and the treatment group 2 mg/kg with 37% and 41%. These two groups showed significantly higher amounts of ear lesions compared to the other treatment groups (*p* < 0.0001). At time point 3, treatment group 4 mg/kg showed the highest amount of ear lesions with 32% followed by the treatment group 6 mg/kg with 19% of ear lesions. Treatment group 4 mg/kg showed a significantly higher amount of ear lesions compared to the control group (4% ear lesions, *p* = 0.0030) and compared to the treatment group 2 mg / kg (7% ear lesions, *p* = 0.0229). All other pairwise comparisons were not significant.

### Tail and ear lesions for the supplementation with MgAsp

**Fig. 4:**
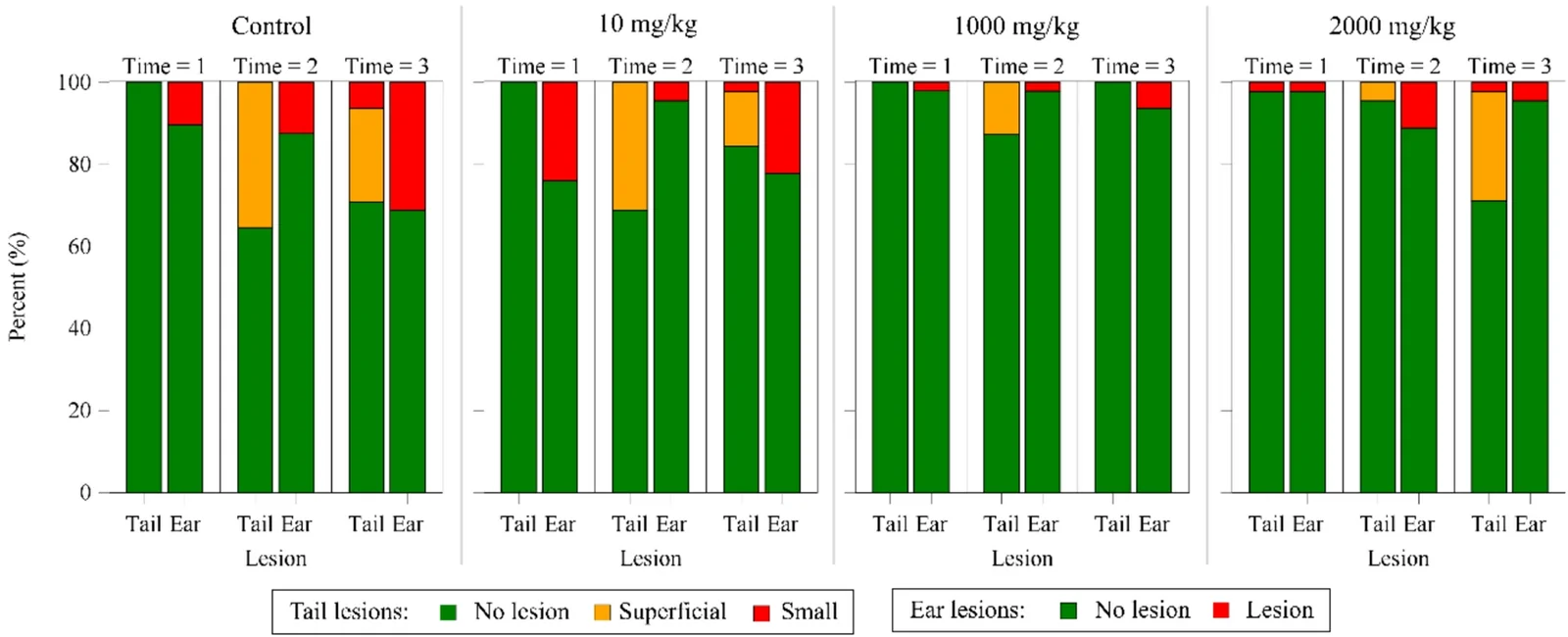
Tail and ear lesions (%) for the supplementation with magnesium aspartate (MgAsp) for the different treatment groups and time points (time point 1: age of the pigs ~ 29 days, time point 2: age of the pigs ~ 7 weeks, time point 3: age of the pigs ~ 9 weeks). Although for statistical analyses tail lesions were binary categorised in no tail lesions and tail lesions, for the descriptive statistic the categorisation no tail lesions, superficial lesions and small lesions was chosen

Figure 4 depicts the percentage of pigs with or without tail or ear lesions across three time points and the different treatment groups for the supplementation with MgAsp. Although the amount of tail lesions was higher compared to batch 1 with supplementation of MgO, over all treatment groups and time points mostly superficial tail lesions could be detected. Only at time point 3, despite the treatment group 1000 mg/kg, small tail lesions up to 6% could be detected, which corresponds to six animals per pen. At time point 1, no significant differences between the treatment groups could be detected. At time point 2, the control group and the treatment group 10 mg/kg showed the highest amounts of animals with tail lesions with 35% and 31%. These amounts were significantly higher compared to the treatment group 2000 mg/kg with only 4% (control vs. 2000 mg/kg: *p* = 0.0012, 10 mg/kg vs. 2000 mg/kg: *p* = 0.0101). All other pairwise comparisons were not significant. At time point 3, the treatment group 1000 mg/kg showed no tail lesions which was significant to all other treatment groups.

The highest amount of ear lesions could be detected at time point 3 for the control group with 31%, so the amounts were lower compared to the supplementation of MgO but more time points and treatment groups were affected. Also, no clear trend over time points could be detected. However, Fig. 4 shows significantly lower amounts of ear lesions for the higher dosages with 1000 mg/kg (2%, *p* = 0.0110) and 2000 mg/kg (2%, *p* = 0.0126) compared to the lower dosage of 10 mg/kg. All other pairwise comparisons were not significant. For time point 2 no significant differences in the amount of ear lesions between the treatment groups could be detected. The amounts of ear lesions range from 2% to 13%. For time point 3, the control group showed with 31% the highest amount of ear lesions followed by the treatment group 10 mg/kg with 22%. However, only the control group showed significantly higher amounts of ear lesions compared to the treatment group 1000 mg/kg (6%, *p* = 0.0190) and compared to the treatment group 2000 mg/kg (4%, *p* = 0.0056). All other pairwise comparisons were not significant.

## Discussion

Overall, it can be noted that all supplementation groups exhibited a decrease in blood magnesium levels after approximately three weeks. This is particularly significant because this is the time when an outbreak of tail biting is often observed in pigs with intact tails [1, 57]. This also fits to the observation of [9] that tail biting might be connected to hypomagnesemia. At time point 3, towards the end of the rearing period, a slight increase in blood magnesium concentration was observed in both batches and treatment groups, except for the control group in batch 1, which did not exhibit this rise. Thus, magnesium supplementation appears effective in this respect. However, whether such supplementation is relevant remains questionable based on this study, as magnesium levels never fell below the reference range indicating no clinical hypomagnesemia as described in [9].

Another important finding is that in batch 2, supplementation of MgAsp, the average magnesium concentration differed significantly among the treatment groups at time point 1, before any magnesium supplementation was provided. This is surprising, considering that all animals originated from the same source and were randomly assigned to groups in accordance with usual farm management. The reason for these differences at this time point remains speculative. Further studies on magnesium supply through sow milk are indicated. While magnesium is a natural component of sow’s milk in general [20], studies show a clear dependency on the mineral nutrition of the sow [28]. Moreover, studies have shown clear benefits of magnesium supplementation on sow reproductive performance [58], whereby the influence on the behaviour of the piglets, especially after weaning, has, to the authors’ knowledge, not been studied so far. Equally important is a closer examination of the reasons for the decline after three weeks in piglet rearing in all treatment groups. A possible explanation is that a secondary deficit occurs at this time after weaning. [54] and [55] have described a potential refeeding syndrome in piglets due to the early weaning which might diminish the potential for mineral uptake in this life phase. This hypothesis requires further investigation in subsequent studies, as a connection to tail biting is plausible [9]. This pilot study on a single farm also points towards this hypothesis of such a connection, as tail lesions followed this trend especially in batch 1. Here, more tail injuries at time point 2 across all treatment groups could be observed, when magnesium concentrations were lowest. By time point 3, a lower percentage of animals had tail lesions, despite in general relatively low observed incidences of tail biting. This trend in pigs with undocked tails has been described in previous studies [1, 57]. It is conceivable that due to early weaning and high productivity, the gastrointestinal system is insufficiently capable of metabolizing the supplied magnesium [19, 25, 26].

Overall, little is known about the effect of magnesium in the age group of rearing pigs [5]. This study therefore is an initial step in closing this knowledge gap and potentially better understanding the pathogenesis of tail biting as a behavioral disorder in the future. A direct link to stress (measured by cortisol) was not demonstrated in this study. Instead, the blood cortisol level followed a rather classic trajectory: The high cortisol level at the beginning of the rearing period can be most likely explained by the stress of weaning and regrouping of unfamiliar animals [34], with pigs typically already establishing a hierarchy at this young age [7]. Such hierarchy establishment is known to be stress-related [34]. A subsequent decrease in cortisol levels to about normal levels for pigs, is expected in light of the literature (e.g. [16]; [21]). Although the methods of blood sample taking differed according to age and size of the piglets – at time point 3 blood samples were collected at the jugular vein and the animals were fixated with a sling while at time points 1 and 2, the animals were turned on their back and blood was collected from the V. cava cranialis, concerning the differences found between time point 1 and 2, this alone cannot be the only explanation for the differences described so the most likely explanation is the connection to weaning and regrouping at time point 1, as described. Nevertheless, as discussed in more detail later on, the results regarding the cortisol level should, however, be interpreted with caution as limitations regarding the sole use of cortisol as stress marker has been highlighted throughout the literature [14, 23, 30] and, moreover, no individual baseline of individual animals could be obtained due to the study design.

Ear lesions exhibited a different trend than tail lesions which has been described in the literature [11, 17]. This underlines the assumption of different pathogeneses for ear and tail biting [9, 29]. A reduction in ear lesions due to magnesium supplementation could not be ascertained from this study.

A limitation of this study was that it was conducted at a farm with extra enrichment and other welfare provisions compared to pig farms only fulfilling the basic legal requirements. Therefore, the level of stress leading to tail and ear biting may be in the low end compared to standard pig farms having pigs with intact tails. Also, in this study tail and ear lesions were studied as outcome-based indicator of tail biting behaviour. Focussing on tail and ear manipulation behaviour (as for example carried out by [56] itself might have been a more sensitive approach.

This study was deliberately designed as a field trial to gain insights relevant to practical applications, as interventions for tail biting must be viable in farming operations to benefit farmers. Laboratory-based research is not helpful in this area, aside from the fact that tail biting cannot be predicted or deliberately provoked [40]. Nonetheless, field trials inevitably introduce limitations for scientific studies [8, 22], as operational priorities take precedence. For example, due to farm constraints, a 10 mg/kg MgO treatment group could not be implemented (though the study results suggest no special effect might have been expected). Additionally, the 8 mg/kg MgO and 40 mg/kg MgAsp were discontinued due to the on-farm conditions, making data collection at time point 3 impossible. Fundamentally, results from a field trial cannot be generalized, meaning the findings cannot be universally applied [41]. Also, this study contained some methodological constraints: number of batches and pens was limited and the pigs were not individually marked making a repeated measure analysis impossible. Blood sampling was carried out as least invasive measure to determine the magnesium content (in comparison to biopsies of organ tissue). As blood samples were then available, the blood was also analysed with regard to cortisol content. It should be noted that the study design was not designed to determine the stress level of the pigs as for stress detection it is usually advised to use a combination of different behavioural and physiological indicators [14, 23, 30] - while here, only results on cortisol level in the blood at the three time points are presented. Moreover, again due to the fact that it was an on-farm trial and animal welfare was prioritized over potential research interests, not from all animals, blood samples became available due to the fact that blood sampling was ceased if the pigs became too agitated or the process for a single pig took too long. In the present study, relatively low numbers of tail lesions were observed and most observations were only superficial lesions. In literature, in the age class of rearing pigs, under conventional circumstances, varying prevalences of 15 to 66% of tail and 11 to 72% of ear lesions have been described while under enriched husbandry conditions (e.g. no mixing, more space, additional manipulableenrichment material) pevalences of 8 to 41% for tail and 0.9 to 40% for ear lesions have been described [11, 15, 27, 29]. It is well conceivable that with more batches a higher incidence or severity of tail bitng might have been observed and clearer conclusions on connections between stress, lesions and magnesium content might have become visible. Moreover, on the present farm, many management intervention strategies known to be beneficial with regard to preventing tail biting (see for example [44] and [53] were, in fact, already in place (e.g. provision of straw as manipulable material, additional enrichment material, additional space, experienced farm staff). In general, tail biting is of multifactorial nature and high complexity [40, 53, 57] and thus this study can only be a small step in contributing to the overall knowledge.

As shown in the inferential statistics, another issue with field trials is the inability to control and clearly separate all effects (since experimental design cannot be as precisely directed as under laboratory conditions). Considerable research remains necessary, and this study contributes as an on-farm case study, although it cannot stand alone given its limitations.

All in all, this study does not show a clear effectiveness of magnesium supplementation in increasing blood magnesium concentration and reducing stress (measured by the physiological stress marker cortisol), tail or ear lesions. This stands in contradiction to literature that has described the effectiveness of magnesium supplementation in reducing physiological stress markers [12, 32, 48, 49]. However, experimental conditions regarding magnesium formulation, dosage, and duration of supplementation varied greatly in these studies, as in studies on magnesium supplementation in pig feeding generally [5]. Standardization of study conditions is urgently needed to develop evidence-based recommendations for practice. In this study, the duration of magnesium supplementation was relatively short (which might be a further limitation of the study design); other studies showing effectiveness used durations of up to 115 days [38]. It should, however, further be noted that the magnesium supplementation in this study also had no noted negative consequences on health or performance of the pigs.

## Conclusion

In this case study under practical on-farm conditions, neither MgO nor MgAsp supplementation during the rearing phase had an effect on the prevalence of tail and ear lesions. Irrespective of type and concentration of magnesium supplementation, there was a consistent decline in blood magnesium at time point 2 (after three weeks in the rearing stable, age of the pigs ~ 7 weeks) supporting the hypothesis made by other authors in the literature that there might be a secondary magnesium deficit potentially due to weaning and gut development. Notably, some treatment groups showed significant differences in the blood magnesium concentration when entering the rearing stable which points towards differences in the supply (or uptake) already in the sow milk. These findings emphasize the need for further research into magnesium metabolism in young pigs and the complex interactions between stress, nutrition, and behavioural disorders such as tail biting.

## Supplementary Information

Below is the link to the electronic supplementary material.

Supplementary Material 1

## Data Availability

The datasets used and analysed during the current study are available from the corresponding author on reasonable request.
