# Supplementary material for: A pilot study on the effect of feed supplementation with magnesium oxide and magnesium aspartate on blood magnesium content and tail lesions in pigs under practical on-farm conditions
Source: Porcine Health Manag. 2026 Jun 11;12:45. doi: 10.1186/s40813-026-00526-0 (PMC13488280; doi:10.1186/s40813-026-00526-0)
Supplement: Supplementary file 1 [file 40813_2026_526_MOESM1_ESM.docx]

**Supplementary material**

**Table S1**: Composition of the commercial basic feed in the two batches (batch 1: magnesium oxide, batch 2: magnesium aspartate) – part 1.

|  | Feed unit | Crude fibre | Crude oils and fats | Magnesium^*^ | Protein |
| --- | --- | --- | --- | --- | --- |
|  | (FU/kg feed) | (%/kg feed) | (%/kg feed) | (g/kg feed) | (%/kg feed) |
| Magnesium oxide |  |  |  |  |  |
| Phase 1 (6-9kg) | 1.216 | 3.15 | 5.53 | 1.18 | 17.55 |
| Phase 2 (9-15kg) | 1.118 | 3.03 | 4.68 | 1.47 | 17.53 |
| Phase 3 (15-30kg) | 1.078 | 3.30 | 3.90 | 1.63 | 18.66 |
|  |  |  |  |  |  |
| Magnesium aspartate |  |  |  |  |  |
| Phase 1 (6-9kg) | 1.216 | 3.61 | 5.53 | 1.18 | 17.55 |
| Phase 2 (9-15kg) | 1.110 | 3.52 | 5.05 | 1.51 | 17.49 |
| Phase 3 (15-30kg) | 1.069 | 3.78 | 4.27 | 1.67 | 18.62 |

*Natural magnesium content in the grain

**Table S2**: Composition of the commercial basic feed in the two batches (batch 1: magnesium oxide, batch 2: magnesium aspartate) – part 2.

|  | Lysin | Methionin | Methionin + Cystein | Treonin | Tryptophan | Valin |
| --- | --- | --- | --- | --- | --- | --- |
|  | (g/kg feed) | (g/kg feed) | (g/kg feed) | (g/kg feed) | (g/kg feed) | (g/kg feed) |
| Magnesium oxide |  |  |  |  |  |  |
| Phase 1 (6-9 kg) | 14.04 | 5.01 | 7.83 | 9.00 | 3.04 | 9.50 |
| Phase 2 (9-15 kg) | 13.54 | 4.44 | 7.44 | 8.62 | 3.13 | 8.95 |
| Phase 3 (15-30 kg) | 14.08 | 4.67 | 7.80 | 8.98 | 3.00 | 9.44 |
|  |  |  |  |  |  |  |
| Magnesium aspartate |  |  |  |  |  |  |
| Phase 1 (6-9 kg) | 14.04 | 5.01 | 7.83 | 9.00 | 3.04 | 9.50 |
| Phase 2 (9-15 kg) | 13.57 | 4.43 | 7.39 | 8.62 | 3.11 | 8.96 |
| Phase 3 (15-30 kg) | 14.11 | 4.66 | 7.75 | 8.98 | 2.98 | 9.45 |

**Table S2**: Comparison of NutriMag MO 600 and NutriMag AS.

| Property | NutriMag MO 600 (MgO, Heavy) | NutriMag AS (Magnesium-L-Aspartate Dihydrate) |
| --- | --- | --- |
| Chemical Formula | MgO | C₈H₁₂MgN₂O₈ · 2H₂O |
| Molar Mass | 40.3 g/mol | 324.5 g/mol |
| Appearance | Fine, white powder | White or nearly white crystalline powder or colorless crystals |
| Solubility | Slightly soluble in water | Easily soluble in water |
| CAS No. | 1309-48-4 | 72231-13-1 |
| EC No. | 215-171-9 | 218-191-6 |
| Purity | ≥ 98.0% (on ignited basis) | 98.0 – 102.0 % (anhydrous basis) |
| Loss on Ignition | ≤ 5.0% (at 900°C) | 10.0 – 14.0 % water content |
| Calcium Oxide | ≤ 1.5% | Not specified |
| Heavy Metals | Lead: ≤ 2 mg/kg, Arsenic: ≤ 3 mg/kg | ≤ 10 ppm |
| Iron | Not specified | ≤ 50 ppm |
| Chloride | Not specified | ≤ 200 ppm |
| Sulfate | Not specified | ≤ 500 ppm |
| Ammonium | Not specified | ≤ 200 ppm |
| pH (2.5% solution) | Not specified | 6.0–8.0 |
| Specific Rotation | Not applicable | +22.0° to +24.0° (anhydrous basis) |
| Tapped Density | 0.25 – 0.65 g/cm³ (10 taps) | Not specified |
| Packaging | 25 kg paper bags | 25 kg drums with polyethylene inner bag |
| Storage Conditions | Store tightly closed in a dry, well-ventilated place, protected from moisture | Store in well-sealed containers at room temperature, away from moisture and direct sunlight |
| Shelf Life | 3 years from the date of manufacture | 3 years from the date of manufacture |
